# Unraveling Polyneuropathy, Organomegaly, Endocrinopathy, Monoclonal Gammopathy, and Skin Changes Syndrome: Diagnostic Challenges and Therapeutic Strategies at a National Tertiary Care Center

**DOI:** 10.7759/cureus.75620

**Published:** 2024-12-12

**Authors:** Kevin Zambrano Zambrano, Julio Martinez Salazar, Jason Cedric Velarde Michaud, Arantxa Montalvo Lopez Gavito, Alexis Zambrano Zambrano

**Affiliations:** 1 Internal Medicine, National Medical Center November 20, Mexico City, MEX; 2 Hematology, National Medical Center November 20, Mexico City, MEX

**Keywords:** bone marrow aspirate, igg lambda, monoclonal gammopathy, plasma cell disorders, poems polyneuropathy

## Abstract

Polyneuropathy, Organomegaly, Endocrinopathy, Monoclonal Gammopathy, and Skin Changes (POEMS) syndrome is a rare paraneoplastic disorder caused by plasma cell proliferation and overproduction of cytokines, particularly vascular endothelial growth factor (VEGF). This complex syndrome affects multiple organ systems and presents with a broad range of clinical and laboratory manifestations, which can complicate both diagnosis and management. Not all components of the acronym are observed in every patient, highlighting the clinical heterogeneity of the condition. The case discussed herein illustrates the diagnostic approach to a patient suspected of having POEMS syndrome.

## Introduction

Polyneuropathy, Organomegaly, Endocrinopathy, Monoclonal Gammopathy, and Skin Changes (POEMS) syndrome, formerly known as osteosclerotic myeloma, is a rare disorder characterized by a plasma cell neoplasm. It is now commonly referred to by its acronym, POEMS. The syndrome typically presents initially as progressive peripheral neuropathy, primarily affecting the lower limbs, and is characterized by axonal damage. The underlying pathophysiology of this clinical presentation is not well defined, but it is believed to be related to the overproduction of vascular endothelial growth factor (VEGF), as well as pro-inflammatory cytokines such as interleukin (IL)-1β, tumor necrosis factor-α (TNF-α), and IL-6 [1,2].

The onset of clinical manifestations is variable, complicating the diagnosis. Consequently, the time from the appearance of symptoms to the diagnosis of POEMS syndrome typically ranges from 13 to 18 months. Another less commonly recognized clinical presentation is referred to by the acronym PEST (Papilledema, Extravascular Volume Overload, Osteosclerotic Lesions, and Thrombosis) [3,4]. The diagnostic criteria for POEMS syndrome are stringent. Two mandatory criteria must be met, along with one major criterion from the three listed and at least one minor criterion from the six available. In this case, the clinical presentation is consistent with the nomenclature of the syndrome [5]. POEMS syndrome is considered a treatable condition. However, the median survival without treatment is approximately 33 months. Therefore, the primary treatment approach aims to prevent the progression of plasma cell proliferation, in addition to alleviating symptoms caused by the multisystem involvement of the disease. Part of the systemic treatment includes immunomodulators and proteasome inhibitors. Even in patients with a poor prognosis, the five-year survival rate is 71% [6,7].

## Case presentation

A 40-year-old female nurse with an 18-year history of smoking and occasional black garlic intake presented with a one-year progressive course of sensory and motor disturbances. The patient initially developed dysesthesias in the pelvic limbs, which progressed to severe distal muscle fatigue and unintentional weight loss of 7 kg over the past three months. Subsequently, she experienced progressive weakness in the upper limbs, resulting in difficulty with grip strength. On admission, clinical examination revealed hepatomegaly and generalized skin hyperpigmentation, and a detailed neurological examination showed significant motor and sensory deficits.

In the neurological examination, muscle strength was scored as 4/5 in the proximal muscles of the upper limbs and 3/5 in the distal muscles of the hands. In the lower limbs, strength was 4+/5 in the proximal muscles and 4-/5 in the left lower limb. Additionally, deep tendon reflexes were present in the upper limbs but absent in the lower limbs. Plantar responses were flexor on the right side and indifferent on the left. Hyporeflexia was also observed in the lower limbs, while Hoffman and Tromner signs were negative.

Electrophysiological studies confirmed a severe demyelinating polyradiculoneuropathy in the pelvic limbs, with moderate involvement in the thoracic limbs. Cerebrospinal fluid analysis showed elevated protein 85.9 mg/dL (reference range: 15-45 mg/dL) without an increase in cell count. In the immunological profile, serum immunoglobulin G (IgG) levels were significantly elevated at 2,834 mg/dL (reference range: 700-1,600 mg/dL). The hormonal evaluation showed elevated thyroid-stimulating hormone (TSH) at 13.7 mIU/L (reference range: 0.4-5.1 mIU/L), along with decreased free T4 and T3 levels of 0.8 ng/dL (reference range: 0.8-1.7 ng/dL) and 1.7 pg/mL (reference range: 2.4-5.6 pg/mL).

The bone marrow aspirate showed unremarkable findings with a low plasma cell count of 5-7%. Morphologically, the plasma cells exhibited normal characteristics without evidence of cytoplasmic inclusions or multinucleation. Other hematopoietic lineages demonstrated preserved morphology with no abnormalities observed.

**Figure 1:**
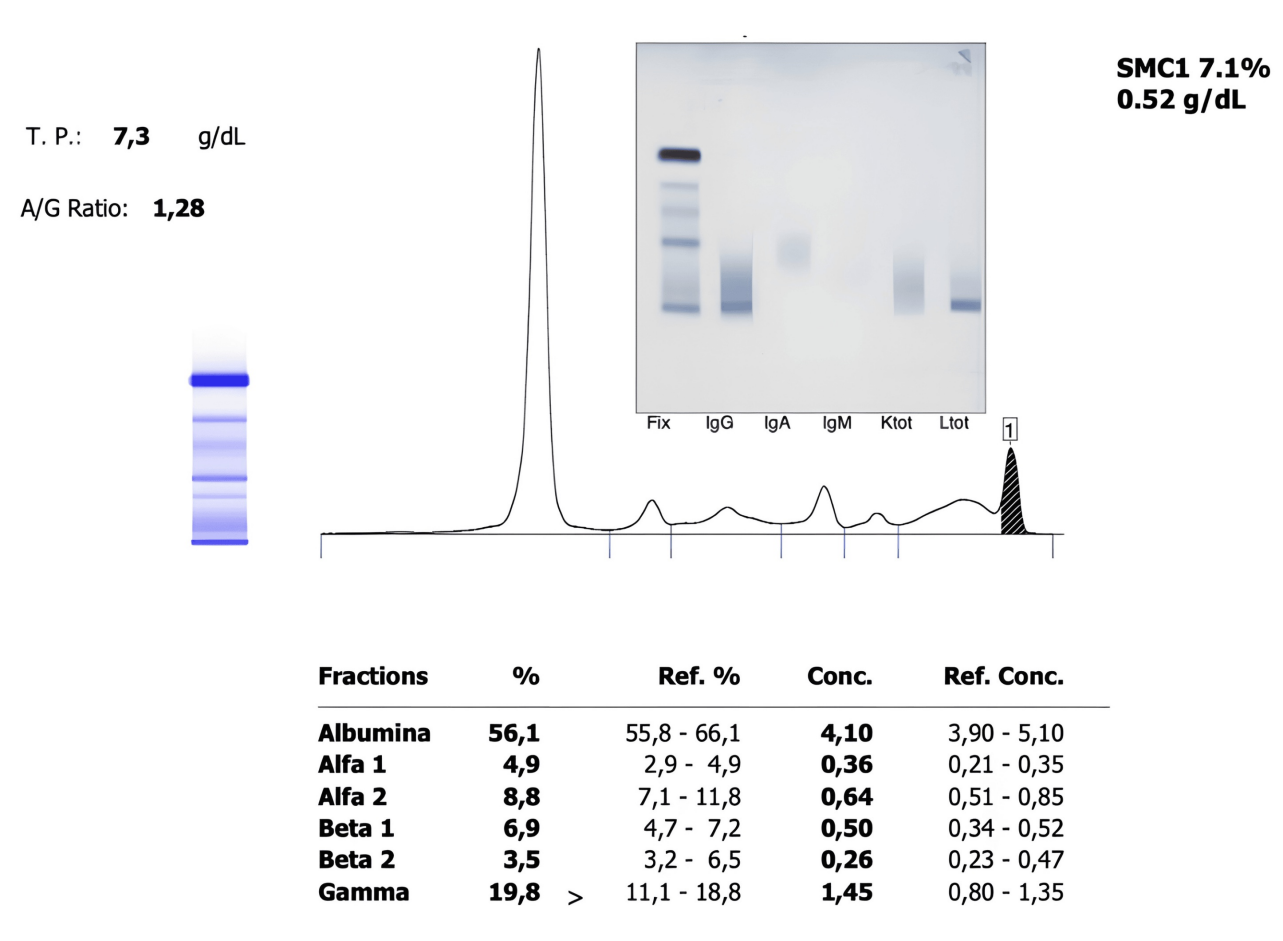
Protein electrophoresis and immunofixation Serum proteins

**Figure 2:**
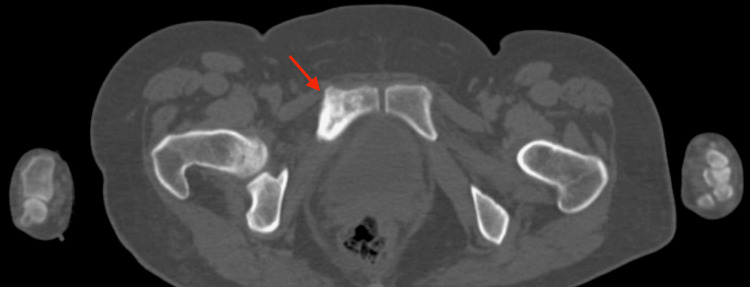
Abdominal computed tomography Blastic bone lesion in the right pubis

**Figure 3:**
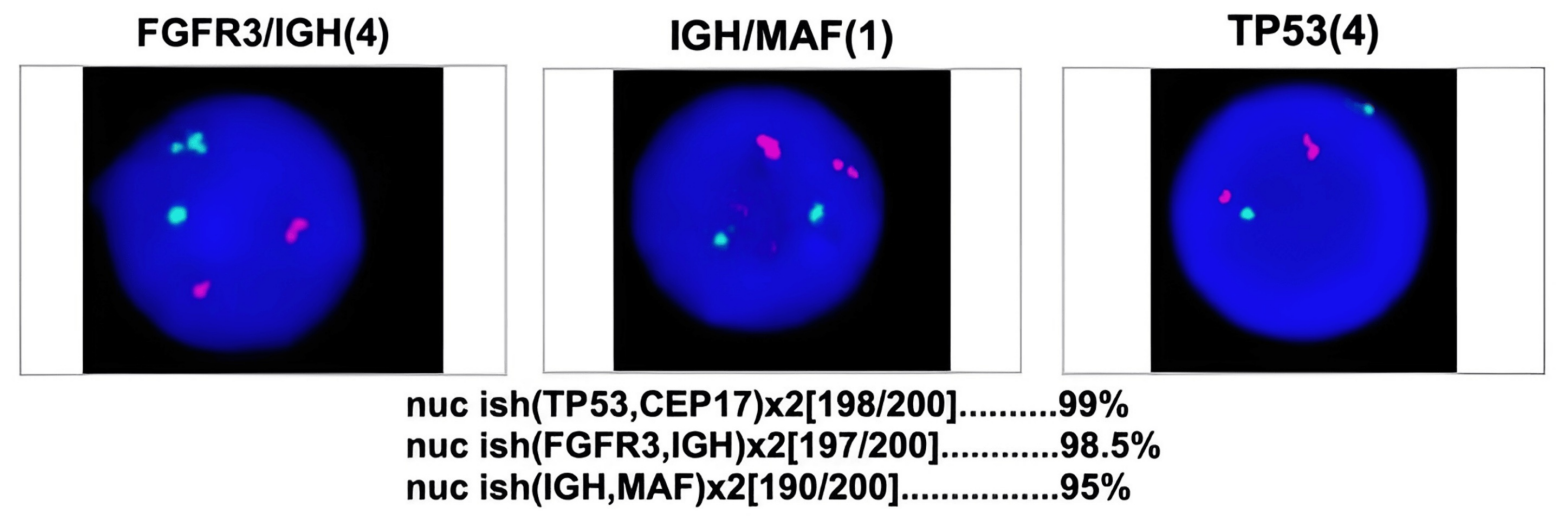
Fluorescence in situ hybridization (FISH) Analysis of bone marrow cells

In imaging studies, serum free light chain analysis revealed elevated free lambda light chains at 198.0 mg/L (reference range: 5-26 mg/L) and a kappa/lambda ratio of 0.17 (reference range: 0.26-1.65), supporting the presence of lambda monoclonal gammopathy (see Figure 1). Abdominal computed tomography identified a blastic bone lesion in the right pubis (see Figure 2). The fluorescence in situ hybridization (FISH) analysis of bone marrow cells showed no high-risk cytogenetic abnormalities associated with hematologic malignancies. The analysis of TP53, FGFR3::IGH, and IGH::MAF was negative for deletions and rearrangements in plasma cells, suggesting a less aggressive profile consistent with the milder forms of POEMS syndrome (see Figure 3). With these findings, a diagnosis of POEMS syndrome was confirmed, and treatment with bortezomib and lenalidomide was initiated to control the gammopathy.

## Discussion

The diagnostic challenge of POEMS syndrome involves differentiating it from other entities, such as multiple myeloma or chronic inflammatory demyelinating polyneuropathy (CIPD). To establish the diagnosis, the mandatory criteria include axonal polyneuropathy and monoclonal proliferation of lambda light chains. Major criteria include the presence of osteosclerotic lesions (Table 1) [3]. The presence of hyperpigmentation, hypothyroidism, and hepatomegaly are minor criteria that are frequently observed in POEMS syndrome [4]. According to a 2020 retrospective observational study of 383 patients with newly diagnosed POEMS syndrome, hyperprolactinemia is the most common endocrine disorder (62.7%), followed by subclinical hypothyroidism (36%) and hypogonadism, which appears to be more common in women than men (48% vs. 22.6%) [8]. Features that have been associated with the worst prognosis are estimated glomerular filtration rate <30 mL/minute/1.73 m^2^, age greater than 50 years, presence of pleural effusion, and pulmonary hypertension [9]. In this case, the patient did not present with any of the high-risk features. Additionally, FISH results were negative for high-risk cytogenetic abnormalities, which allowed the selection of a standard treatment regimen. Bortezomib, a proteasome inhibitor, helps reduce the proliferation of abnormal plasma cells and has been demonstrated in multiple clinical trials to be a well-tolerated and highly effective first-line treatment regimen for POEMS syndrome, while lenalidomide, an immunomodulator, enhances immunity and promotes the destruction of malignant cells and has been studied for both induction and maintenance therapy [10,11]. In cases with high-risk genetic alterations, such as TP53 deletion or high-risk translocations, a more intensive approach is recommended, which may include daratumumab or carfilzomib in combination with conventional chemotherapy or even an autologous stem cell transplant [5]. Follow-up of patients is usually made every three to four months. There is no consensus on how to define response, but clinical trials usually define response in complete hematological response (undetectable M protein by serum and urine immunofixation electrophoresis), neurological response (reduction of at least one ONLS scale score compared with baseline), and serum VEGF response (complete: VEGF < 600 pg/mL; partial: VEGF improved by at least 50% compared with baseline) [10].

**Table 1:** Diagnostic criteria for Polyneuropathy, Organomegaly, Endocrinopathy, Monoclonal Gammopathy, and Skin Changes (POEMS) syndrome

Mandatory criteria (both must be present)	Major criteria (at least one must be present)	Minor criteria (at least one must be present)	
Polyneuropathy	Sclerotic bone lesions	Papilledema	Thrombocytosis/polycythemia
	Castleman disease	Skin changes	Edema/ascites and/or pleural effusion
Monoclonal plasma cell-proliferative disorder	Vascular endothelial growth factor elevation	Splenomegaly, hepatomegaly, or lymphadenopathy	Endocrinopathy (adrenal, thyroid, prolactin, parathyroid, pancreas, gonadal)

## Conclusions

Early diagnosis of POEMS syndrome relies heavily on high clinical suspicion due to its diverse presentation and overlap with other conditions. Key findings, such as axonal polyneuropathy, monoclonal lambda light chain gammopathy, and associated clinical features, should raise suspicion for POEMS. Once diagnosed, evaluating the patient’s genetic profile is crucial, as the absence of high-risk cytogenetic abnormalities allows for a standard treatment approach with bortezomib and lenalidomide. In contrast, cases with high-risk features should be managed with more intensive strategies, such as daratumumab or stem cell transplantation. The combination of clinical suspicion, genetic analysis, and personalized treatment is essential for optimal management of POEMS syndrome.
